# Increased brain ^1^H-MRS glutamate and lactate signals following maximal aerobic capacity exercise in young healthy males: an exploratory study

**DOI:** 10.5114/biolsport.2023.118335

**Published:** 2022-09-15

**Authors:** Maciej Świątkiewicz, Stefan Gaździński, Michał Madeyski, Bartosz Kossowski, Józef Langfort, Piotr Bogorodzki, Ewelina Zawadzka-Bartczak, Katarzyna Sklinda, Jerzy Walecki, Paweł Grieb

**Affiliations:** 1 Department of Experimental Pharmacology, Mossakowski Medical Research Institute, Polish Academy of Sciences, Warsaw, Poland; 2 Military Institute of Aviation Medicine, Warsaw, Poland; 3 Laboratory of Brain Imaging, Nencki Institute of Experimental Biology of Polish Academy of Sciences, Warsaw, Poland; 4 Faculty of Electronics, Warsaw University of Technology Warsaw, Poland; 5 Institute of Sport Sciences, The Jerzy Kukuczka Academy of Physical Education, Katowice, Poland; 6 Small Animal Magnetic Resonance Imaging Laboratory, Mossakowski Medical Research Centre, Polish Academy of Sciences, 02-106 Warsaw, Poland; 7 Interinstitute Laboratory of New Diagnostic Applications of MRI (CNSLab), Nalecz Institute of Biocybernetics and Biomedical Engineering, Polish Academy of Sciences, 02-109 Warsaw, Poland

**Keywords:** Glutamate, Lactate, Magnetic resonance spectroscopy, Exercise, Brain, Occipito-parietal grey matter

## Abstract

Physical exercise involves increased neuronal activity of many brain structures, but 1H-MRS investigations on the effects of human brain glutamate (Glu) concentrations on acute exercise have been sparse. Previous studies consistently found increases in brain lactate (Lac) concentrations following graded exercise up to 85% of the predicted maximal heart rate. However, the reported effects on brain concentrations of glutamine and glutamate were not consistent. This study aimed to determine the effect of acute intense graded maximal exercise on 1H-MRS signals related to concentrations of Glu, glutamate+glutamine (Glx), and Lac. Young adult males were randomly divided into two groups and subjected to 1H-MRS when resting (NE) or shortly after cessation of the intense graded exercise intended to pass the anaerobic threshold (E). 1H-MRS spectra were acquired from the large voxel encompassing the occipito-parietal cortex only once. Estimates of Glu, Glx, and Lac concentrations were calculated in institutional units by normalizing to a spectroscopic signal originating from creatine-containing compounds (Cr). Concentrations of Glu, Glx, and Lac were respectively 11%, 12.6%, and 48.5% higher in E than in NE (p < 0.001). The increased brain Lac signal in the exercising group indicated that in our experiment, vigorous exercise resulted in passing the anaerobic threshold and lactate apparently entered the brain. Concomitantly glutamate-related resonance signals from the vicinity of the occipito-parietal cortex were significantly increased; physiological mechanisms underlying these phenomena require further study. Future studies should evaluate whether the normalization rate of these concentrations is a marker of general physical fitness.

## INTRODUCTION

Exercise is a simple environmental intervention that profoundly modifies brain function by inducing a cascade of molecular and cellular processes that support brain plasticity. The effect on the exercise-induced plasticity of the brain may be driven from impulses generated outside the brain. The best-recognized evidence presented in human and animal studies shows an association between contraction-induced changes in working muscles of metabolic products and central nervous system (CNS) function by stimulation of group III and IV muscle afferents [1, 2, 3, 4]. Moreover, afferent signals from the lungs (increased ventilation) and the heart (increased heart rate) were found to affect brain function during exercise [5]. The onset of effects of exercise on the brain is rapid and evolves over time [6]. Initially, in response to exercise, neurotransmitter levels change, followed by upregulation of growth factors in the brain [e.g., brain-derived nerve growth factor (BDNF)] and the genesis of new neurons in the hippocampus [7, 8]. Acute normobaric hypoxia does not affect the simultaneous exercise-induced increase in circulating brain-derived neurotrophic factor (BDNF) and glial derived neurotrophic factor (GDNF) in healthy young men [9]. It is well documented that dopaminergic and serotonergic pathways play a pivotal role in movement regulation, and their activity is strongly influenced by exercise [10] and would provide an input to the central governor, defined as the functional structure of the brain responsible for regulation of movement [11, 12].

Glutamate (Glu) is the main excitatory neurotransmitter in the mammalian central nervous system, and 60–80% of total energy consumption in the non-stimulated cerebral cortex is used by processes supporting glutamatergic neurotransmission [13]. A key part of the glutamatergic neurotransmission system is the so-called glutamate-glutamine cycle (Glu-Gln cycle), believed to efficiently recycle Glu released to synaptic clefts between synapses and presynaptic terminals. The cycle involves uptake of Glu from synapses by astrocytes, intra-astrocytic conversion of Glu to Gln, transport of Gln from astrocytes to neurons, and intraneuronal conversion of Gln back to Glu [14, 15].

Magnetic resonance spectroscopy (MRS) in vivo provides opportunities for noninvasively probing certain aspects of glutamatergic neurotransmission. Changes in brain proton magnetic resonance signals of these substances are only indirectly related to the rates of their metabolism, but certain information concerning the function of the glutamatergic neurotransmission system can be inferred from such observations. Although only a fraction of MRS-detectable glutamate is involved in neurotransmission, it has been argued that differences in MRS-visible glutamate, such as between healthy persons and patients suffering from mood disorders or schizophrenia, are likely related to changes in glutamatergic neurotransmission [16, 17].

Physical exercise involves markedly increased neuronal activity of many brain structures [18, 19], but investigations of the effects of acute exercise on estimated brain levels of Glx or Glu in humans have seldom been performed and the results obtained are not consistent. In a 1H-MRS study employing a 1.5T MR system, Maddock et al. [20] observed an 18% increase in the Glx signal from the visual cortex following graded exercise to approximately 85% of the predicted maximal heart rate. In a further study from the same laboratory, performed using a 3T MR system [21], an increase in glutamate resonance acquired following exercise from the visual cortex was confirmed, and a similar increase was noted in the anterior cingulate cortex. The authors interpreted these results as consistent with the exercise-induced expansion of the cortical pool of glutamate, seemingly in agreement with a marked increase in whole-brain nonoxidative glucose consumption [22]. However, other investigators [24], who employed a similar exercise paradigm but a 7T MR system and recorded 1H-MRS spectra from the occipital cortex, did not find a significant change in Glu or glutamine (Gln) following similar vigorous exercise when the metabolites were normalized to creatine. However, when the concentrations were scaled to a water signal, a decrease in both Glu and Gln was observed.

We hereby present the results of an exploratory study performed to evaluate the differences between brain 1H-MRS signals acquired from the brains of healthy young human volunteers at rest and following short-term acute exercise. We aimed to find out whether acute graded vigorous exercise continued until the subject reaches a predefined maximum heart rate limit is reflected in Glu-dependent resonance signals from the brain. We chose to collect H-MRS spectra from a large voxel encompassing the occipito-parietal cortex and vicinity because these areas are expected to be activated during exercise. The parietal cortex becomes activated with a diverse range of stimuli and tasks, including motion processing [24], and the occipito-parietal cortex is activated and becomes thickened following repeated exercise [25].

The other 1H-MRS-detectable effect of acute intense exercise in humans is the increase of the lactate (Lac) signal from the brain, which has been reported independently of whether the Glu signal was found to be increased [20, 21] or decreased/unchanged [23]. In the present experiments recording the brain lactate signal was not a primary goal, but it was performed in order to confirm the comparability of our experimental paradigm with those in the previous brain 1H-MRS exercise studies. We expected higher concentrations of Glu, Glx, and Lac in the exercising group (E) than in the resting (non-exercising, NE) group.

## MATERIALS AND METHODS

### Subjects

Twenty-eight young male candidate cadets taking the Polish Air Force University entrance exam volunteered for the study. They were aged 17–19 years, with a height of 177.8 ± 8.0 cm and a body weight of 76.5 ± 7.6 kg. As military cadets, participants had high physical fitness. They were randomly divided into two groups: collected at rest (NE group) and shortly after the end of the exercise in the exercise (E group). All participants had a current valid medical examination that excluded chronic diseases such as diabetes or epilepsy. They stated that for at least one month before testing they did take medications or dietary supplements. Participants were informed of the nature of the investigation, with a clear statement of the objective of the research and possible risk. They could withdraw at any time. The experimental protocol conformed to the principles presented in the Declaration of Helsinki and was approved by the ethical committee of the Military Institute of Aviation Medicine.

### Exercise testing protocol

The participants were instructed to abstain from alcohol, coffee, tea, and strenuous exercise for at least two days before the study. Visits to the laboratory began at 10:00 AM after all-night sleep (i.e., the participants were not tired), 2–3 hours after a light breakfast composed of 50–60% carbohydrates, 15–20% protein, and 22–30% fat. The participants were randomly assigned to the non-exercising (control) group (NE, n = 14) and the exercising group (E, n = 14). For members of the exercising group, the MRI session started within 10 min after the end of the exercise.

The progressive exercise test was performed on a treadmill according to Mortara Qstress System guidelines (Mortara Instrument, Inc., Milwaukee, Wisconsin USA). Here we employed a routine procedure required by the Polish Ministry of National Defence for testing candidates for fighter pilots. Briefly, in the pre-exercise phase study, participants underwent short forced hyperventilation in the standing position (breaths as deep and rapid as possible for less than 1 min). During this phase, the Q-Stress system acquires ECG data to develop the patient’s cardiac template used for heart rate limit calculation, ST-segment analysis, and possible arrhythmia detection. Three minutes later, a 1-minute warm-up was initiated with no treadmill inclination set at a velocity of 1.6 km/h, followed by the SIMULATOR treadmill protocol of graded exercise (Table 1). The test is continued until the subject reaches the age-dependent maximum heart rate limit, which is set as (220-age) beats per minute, as recommended for cardiovascular stress testing that uses exercise [26]. All participants fulfilled the conditions below, and none of the tests were interrupted for the following reasons: exhaustion; diastolic blood pressure increase (DBP) > 120 mmHg; systolic blood pressure (SBP) increase > 260 mmHg; sustained drop in SBP; clinical manifestation of intense typical chest pain; depression of the ST segment > 3 mm; elevation in the ST segment > 2 mm in the lead without the presence of the Q wave; complex ventricular arrhythmia; the appearance of sustained supraventricular tachycardia, atrial tachycardia, or atrial fibrillation; 2^nd^- or 3^rd^-degree atrioventricular block, signs of left ventricular failure; a failure in the monitoring or recording systems, or both. The presence of typical symptoms or symptoms of straightened or descending depression in the ST segment > 1.0 mm or ascending depression of the ST segment > 1.5 mm, 0.08 seconds from the J point, or even an elevation in the ST segment 1.0 mm, characterized a positive test. The test was considered successful if the patient reached at least 100% of the maximum heart rate recommended (220-age). Hypertension reactive to the effort was defined as SBP levels > 220 mmHg or an elevation of 15 mmHg or more in DBP, or both. Moreover, the indirect measure of maximum oxygen consumption (VO_2_max), in METS, was automatically calculated by the software using the formula of the American College of Sports Medicine.

**TABLE 1:** SIMULATOR protocol: duration, velocity, and incline of the treadmill at every stage.

Stage	Time of duration	Velocity	Incline
1	1:00 min	4.0 km/h	12%
2	1:00 min	5.3 km/h	14%
3	1:00 min	6.8 km/h	14%
4	1:00 min	8.0 km/h	16%
5	1:00 min	8.9 km/h	18%
6	1:00 min	9.5 km/h	20%
7	1:00 min	11.1 km/h	22%
8	1:00 min	12.1 km/h	22%

### Magnetic resonance sessions

**FIG. 1:**
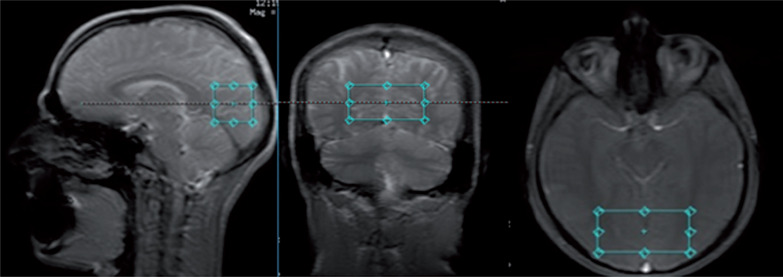
Placement of the volume of interest (30 × 30 × 60 mm^3^) in the area encompassing the occipito-parietal cortex of both hemispheres.

In this cross-sectional study, MR studies were performed with a 3T GE Discovery 750w scanner with a 70 cm wide bore, using a body transmit coil for excitation and the eight-channel receiver coil (description available in the GE catalogue). Each MR session consisted of structural imaging performed for voxel placement and localized single-voxel proton MR spectroscopy. The structural scan was obtained using steady-state fast spin echo (SSFSE) with 3 slices in each orthogonal plane, with the following parameters: repetition time/echo time (TR/TE) = 6.412/2.1 ms, duration 30 s. Spectroscopic data were acquired with a PRESS PROBE-P single voxel sequence (TR/ TE = 1500/35 ms and TR/TE = 1500/288 ms, bandwidth = 2367 Hz, 128 acquisitions, duration 3 min 48 s), with chemical shift selective (CHESS) water suppression. The spectra were acquired with a short echo time (35 ms) to measure Glu and Glx and with a long echo time (288 ms) to measure Lac. Both spectra were acquired within the same volume of interest (VOI, 30 × 30 × 60 mm = 54 cm^3^), which was placed in the vicinity of the occipitoparietal grey matter area (Fig. 1). A large VOI was used to allow for monitoring of small signals from Glu and Glx within reasonable time.

Unsuppressed water signals were acquired for each sequence for eddy current compensation and phase correction. 4.096 complex points were acquired on the spectrum.

### Spectra processing

**FIG. 2:**
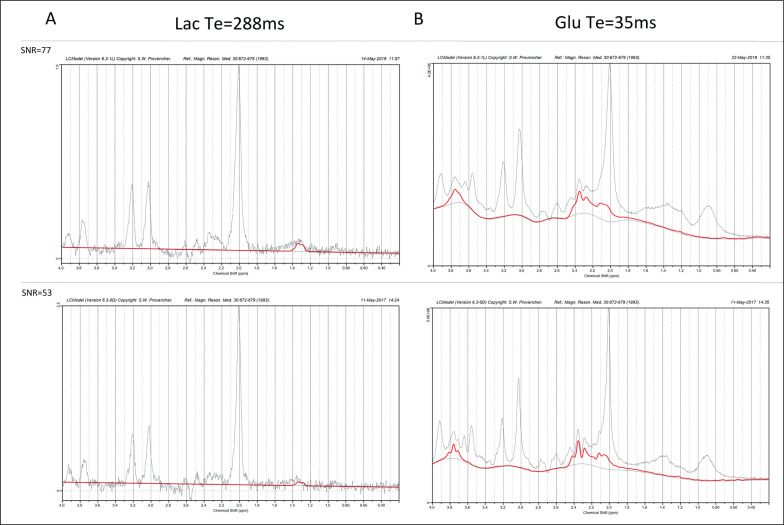
Representative spectra (black line; the best SNR = 77 (upper graph) and the worst SNR = 53 (lower graph)) and LCModel approximation of metabolite signals (red line). A: Lac, TE = 288 ms, B: Glu, TE = 35 ms, Average CRLB: TE = 288 ms, Lac = 18.4, TE = 35 ms Glu = 4.7.

Metabolite concentrations were estimated using the LCModel software [27], version 6.3.-1M. The default LCModel pre-processing pipeline was utilized, i.e., the sum of squares channel combined and phase cycle accumulation. The analysis window used with LCModel was 4.0 to 0.2 ppm. An example of the LCModel approximation of metabolite concentrations superimposed on the representative spectra obtained from the occipito-parietally localized VOI is shown in Fig. 2.

The ‘standard’ basis set for the 3T PRESS sequence with TE 35 ms or 288 ms was used, containing the following metabolites: alanine (Ala), aspartate, (Asp), creatine (Cr), phosphocreatine (PCr), glycerophosphocholine (GPC), phosphocholine (PCh), γ-aminobutyric acid (GABA), glucose (Glc), glutamine (Gln), glutamate (Glu), glutathione (GSH), lactate (Lac), myo-Inositol (Ins), N-acetyl aspartate (NAA), N-acetyl aspartate glutamate (NAAG), scyllo-Inositol (Scyllo) and taurine (Tau). The simulated basis set was provided by S. Provencher on request. Specified timings and ideal pulses were used for gamma simulation. The signal-to-noise ratio (SNR) was derived from the LCModel. Relative standard deviation estimates of the resonance signals (Cramér-Rao lower bounds, CRLB) were used to establish the criterion for further analysis. Resonance signals with mean CRLB ≤ 15% for metabolites and ≤ 30% for Lac (excluding macromolecules) were identified and used to obtain estimates of their respective concentrations.

Metabolite signals in the acquired spectrum were scaled to the signal of the sum of creatine and phosphocreatine (tCr) [28]; i.e., the concentrations were measured in institutional units, not in moles/ grams of the neurotransmitter of interest per unit volume of the brain. The term concentrations will refer to concentrations measured in institutional units throughout the manuscript. Spectral quality was comparable across groups (see Table S1).

### Statistical analyses

The GraphPad Prism for Windows ver. 6.04 software package (Graph-Pad Software, San Diego, CA, USA) was used for statistical assessment of the data. The normality of distribution was assessed with D’Agostino–Pearson omnibus and Shapiro–Wilk normality tests. Because estimates of metabolite concentrations in the E group did not follow a normal distribution, non-parametric tests were used for data analysis. No correction for multiple comparisons was applied. The significance of differences between the data collected at rest (NE group) and exercise (E group) were tested using the Mann-Whitney test. Differences between groups were considered significant when p ≤ 0.05.

## RESULTS

**FIG. 3:**
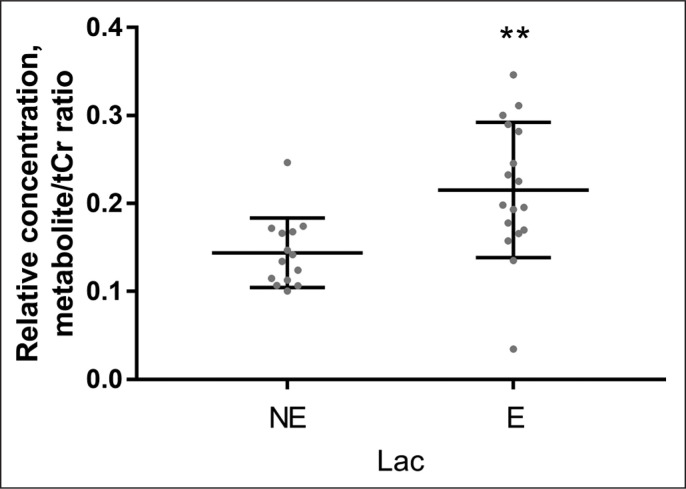
Relative concentrations of Lac obtained from data recorded with TE = 288 ms, in the non-exercising group (NE) and in the exercising group (E). ****P < 0.0001.

**FIG. 4:**
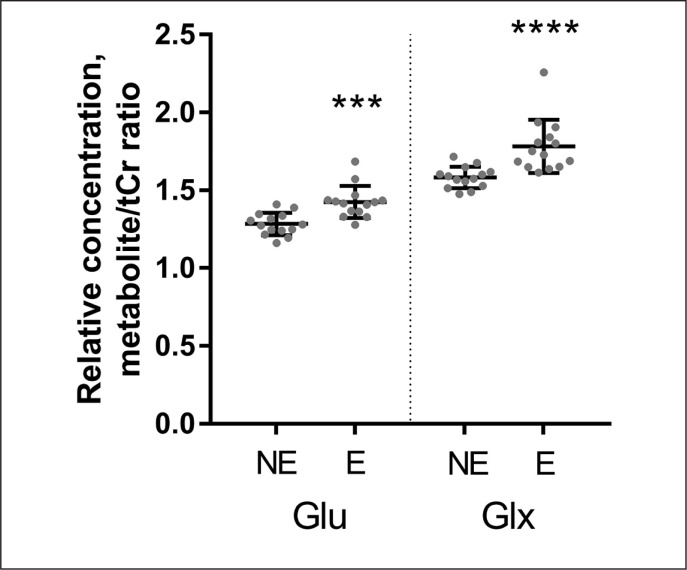
Relative concentrations (mean values and standard deviations) of Glu and Glx obtained from data in the non-exercising group (NE) and in the exercising group (E). Individual values of metabolite concentrations scaled to total creatine (TE = 35 ms). *P < 0.0001.

Quantitative data concerning Glu Glx and Lac scaled tCr are presented on the graphs as individual data points and mean values for the NE and the E groups (Figs. 3–4). When 1H-MRS spectra recorded at rest and after exercise were compared, signals of Glu, Glx, and Lac were higher in the exercising group than in the resting group. Following exercise, the mean Lac was 49.5% higher than at rest (p = 0.001); the mean Glu was 11.0% higher than at rest (p = 0.0001); the mean Glx was 12.6% higher than at rest (p = < 0.0001). All measured metabolites (mean values and standard deviation) are presented in Table 2.

**TABLE 2:** Average relative concentrations of measured metabolites for non-exercising group (NE) and exercising group (E).

X/tCr	NE Group		E Group		P value

M	SD	M	SD
Lac	0.14	0.04	0.22	0.08	0.001
Glu	1.28	0.07	1.42	0.10	0.0001
Glx	1.58	0.07	1.78	0.17	< 0.0001
NAA	1.42	0.05	1.49	0.11	0.08
NAA+NAAG	1.56	0.10	1.64	0.09	0.08
GPC+PCh	0.20	0.02	0.21	0.03	0.43
Ins	0.78	0.04	0.78	0.04	0.82
GPC	0.18	0.05	0.18	0.05	0.43
GSH	0.16	0.04	0.17	0.04	0.81
NAAG	0.14	0.06	0.15	0.05	0.94
Tau	0.19	0.04	0.23	0.08	0.01

Note: values are mean and standard deviations, X/ tCr - Metabolite scaled to the signal of the sum of creatine and phosphocreatine, Lac - Lactate, Glu - glutamate, Glx - glutamate and glutamine, NNA - N-acetylaspartate, NAA+NAAG - N-acetylaspartate and N-acetylaspartylglutamate, GPC+PCh - glycerophosphocholine and phosphocholine, Ins - inositol, GPC - glycerophosphocholine, GSH - glutathione, NAAG - acetylaspartylglutamate, Tau - Taurine.

## DISCUSSION

The major finding of the present study is that in young male volunteers, 1H-MRS signals of Glu and Glx recorded from the large voxel located in the vicinity of the occipito-parietal cortex shortly after the exercise were significantly higher than those recorded at rest. Our data confirm the findings of Maddock et al. [20], who observed an increase in Glx in the visual cortex of healthy volunteers following short strenuous exercise and attributed it to the exercise-induced expansion of the cortical pool of glutamate.

During recent years a body of evidence has accumulated indicating that both acute bouts of exercise and regular training contribute to the activation of numerous adaptive mechanisms of the human body. These exercise/training-induced adaptive changes increase the improvement in both health and exercise performance in sedentary subjects and athletes. The extent of these changes is primarily determined by the type of exercise/training, exercise/training loads, and the sports level of athletes [29, 30]. In recent years, a pivotal role in the regulation of these changes has been assigned to the brain, particularly the prefrontal cortex, which takes part in the regulation of many executive functions to prepare humans for situations demanding high levels of working memory, attention, and cognitive flexibility [31, 32]. Thus, the main contribution of this study was to provide new and relevant data on the effect of maximal exercise on glutamate and lactate signals in young, healthy men, which may be involved in the modulation of the above-mentioned functions in response to exercise. We chose these variables for our research because glutamate is one of the main neurotransmitters in the brain, and lactate, especially during exercise, is taken up by the brain and can significantly modify its functions [33]. Moreover, the role and contribution of glutamate is the least studied in response to exercise among the neurotransmitters.

According to the model presented by Fonnum and Hassel [34] there are four Glu pools in the brain: the transmitter pool, the glial pool, the GABA precursor pool, and the metabolic pool. These authors pointed out that different pools are housed by different compartments. The metabolic Glu pool resides in the neuronal bodies; the glial pool resides in the glial cells; the transmitter and GABA precursor pools reside in the presynaptic terminals, excitatory and inhibitory, respectively. Whereas the average brain Glu concentration (determined by chemical methods) is approximately 10 mM [35], its concentration in glutamatergic synaptic terminals is much higher. Presynaptic glutamate is stored in synaptic vesicles, from which it is released upon stimulation [36], and Glu concentration in the vesicles is thought to reach 100 mM [37].

The 1H-MRS technique provides information on the average Glu concentration in the chosen volume of interest, and the aforementioned glutamate pools in the brain are usually not mentioned in interpretations of the spectroscopic data. When an increase in the average Glu pool in brain tissues is detected using 1H-MRS technique, information is provided neither about relative sizes of the various Glu pools nor about which pool/s of Glu is/are responsible for the overall change detected. However, the concentrations of Glu and Gln are higher in the brain than in any other tissue [38]. Considering that 85% of the energy consumed by the resting brain is used for support of glutamatergic neurotransmission, we may be rather certain that an increase in the average brain Glu content during exercise is related to the enhancement of excitatory neurotransmission and neurotransmitter Glu recycling through Gln. The other important functions of Glu in the brain served by its metabolic pool are nitrogen buffering (ammonia detoxification) and providing an input into the tricarboxylic acid cycle (TCA) [39]. However, ammonia is released from the working muscles to blood; in rat experiments, the requirement for NH_3_ removal from the brain during exercise was increased, and glutamine synthesis was stimulated as a mechanism of detoxication, resulting in brain Glu decrease [40]. Also, supporting enhanced neurotransmission is the only additional work performed in the brain during exercise compared with rest, and it would be very impractical to use Glu to fuel the TCA cycle. Considering all the above, it seems rather certain that the exercise-induced expansion of the total brain Glu pool is caused mostly, if not totally, by the expansion of the Glu pool serving neurotransmission.

The other finding of the present study concerns the exercise-induced increase in the lactate signal. This observation is also in agreement with the findings of Maddock et al. [20, 21], who attributed increased Lac resonance to the entry of lactate from the blood to the brain during exercise. Such an interpretation agrees with the prevailing view that during intense exercise, when lactate is released into the blood by the working muscles, blood lactacidaemia makes it available to the brain, which switches from a net producer to a net importer of lactate [41]. An increase in 1H-MRS Lac signal recorded from the brain has been reported following sodium lactate infusion in healthy volunteers [42]; this result indicates that increased brain activity is not a prerequisite for lactate entering the brain from the blood. On the other hand, in an fMRI experiment, a significant increase of the lactate signal (by an average of 8.6%) was observed in the motor cortex following the standard finger-tapping paradigm [43]. It is possible that a part of the increased Lac signal from the brain following the exercise bout reflects the intensification of the aforementioned increase of glucose metabolism to lactate and subsequent anaplerotic metabolites, including glutamate. However, in our data, there was no correlation between Glu and Lac signals, the correlation coefficients being r = 0.004 and r = 0.06 for resting and post-exercise groups, respectively (data not shown).

Our study has several limitations. Some of them are typical for clinical studies employing 1H-MRS. Clinical MR scanners are frequently employed to estimate Glu concentrations in the human brain, but values obtained vary among the studies. For example, average values of Glu concentrations in predominantly grey matter (GM) of adult human brain estimated from H-MRS spectra acquired using 1.5, 3 or 7T magnets published prior to 2003 ranged from 7.1 to 12.5 mmol/L; see Table 5 in [44]. More recently, a comparison of 1H-MRS of 3 regions of the human brain at 3 and 7 T, using the same subjects and similar methodology, revealed significant differences in the estimates of the concentration of some metabolites, e.g. Glx [45]. Although the aforementioned dispersion of the data seems to indicate a rather high inaccuracy of the 1H-MRS technique as a tool for estimation of brain metabolite concentrations, such a situation is not unusual in neurochemistry. In a landmark study concerning the *in vivo* neurochemical profile of rat brain [46], estimates of metabolite concentrations obtained from ultra-short echo time 1H-MRS were compiled with data obtained using non-resonance analytical techniques, published in the neurochemical literature, including reference textbooks. For Glu, the resonance-derived estimate was 8.3 mmol/g brain, while the classical data ranged from 7.3 to 12.5 mmol/g brain. This comparison shows that resonance-derived estimates of brain metabolite concentrations may not be less accurate than the data obtained with other techniques.

Further limitations are related to the exploratory character of our study. We chose to compare the proton magnetic resonance data collected at rest and shortly after the end of a bout of strenuous exercise from a large voxel located around the occipito-parietal cortex area and subjected to minimal processing. Our main aim was to determine whether the estimates of Glx and Glu concentration are different between the resting and post-exercising group, while the accuracy of the measurements was of secondary importance. We did not perform frequency drift correction, but magnetic resonance spectra acquisitions lasted only 4 min, and over this time, no significant drift is expected [47]. Also, we did not record non-averaged data and did not apply a correction for actual volume fractions of grey matter, white matter, and fluid spaces. We assumed that, since the study participants were highly homogeneous with respect to age and general health conditions, the composition of voxels from which the spectra were collected are also highly similar in all of them. In agreement with this expectation, there was no difference between the NE and the E group with respect to SNR, FWHM (full width at half maximum), and CRLB values for all quantifiable metabolites. When the estimates of metabolite concentrations were obtained with LCModel, statistically significant differences between the NE and the E group were observed for Glx, Glu, and Lac, and also taurine. However, the taurine signal displayed a high CRLB value, and the difference disappeared after correction for multiple comparisons was applied.

## CONCLUSIONS

In summary, our exploratory experiment showed that resonance signals of Lac, Glu, and Glx recorded from the large voxel around the occipito-parietal cerebral cortex of young, healthy volunteers after a single episode of vigorous exercise were higher than those recorded at rest. The present data do not provide an explanation for the physiological mechanisms underlying these effects. In particular, further studies are needed to understand the relation between the increased rate of glutamate neurotransmission and the concomitant increase in glutamate-dependent resonance signals from the brain.

## Supplementary Material

Increased brain ^1^H-MRS glutamate and lactate signals following maximal aerobic capacity exercise in young healthy males: an exploratory study

## References

[1] Haouzi P, Hill JM, Lewis BK, Kaufman MP. Responses of group III and IV muscle afferents to distension of the peripheral vascular bed. J Appl Physiol (1985). 1999;87(2):545–53. doi: 10.1152/jappl.1999.87.2.545. 10444611

[2] Hayes SG, McCord JL, Koba S, Kaufman MP. Gadolinium inhibits group III but not group IV muscle afferent responses to dynamic exercise. J Physiol. 2009;587(Pt 4):873–82. doi: 10.1113/jphysiol.2008.164640. 19103679 PMC2669976

[3] Jankowski MP, Rau KK, Ekmann KM, Anderson CE, Koerber HR. Comprehensive phenotyping of group III and IV muscle afferents in mouse. J Neurophysiol. 2013;109(9):2374–81. doi: 10.1152/jn.01067.2012. 23427306 PMC3652219

[4] Amann M, Sidhu SK, Weavil JC, Mangum TS, Venturelli M. Autonomic responses to exercise: group III/IV muscle afferents and fatigue. Auton Neurosci. 2015;188:19–23. doi: 10.1016/j.autneu.2014.10.018. 25458423 PMC4336599

[5] Lam E, Greenhough E, Nazari P, White MJ, Bruce RM. Muscle metaboreflex activation increases ventilation and heart rate during dynamic exercise in humans Exp Physiol. 2019;104(10):1472–1481. 31206823 10.1113/EP087726

[6] Basso JC, Suzuki WA. The Effects of Acute Exercise on Mood, Cognition, Neurophysiology, and Neurochemical Pathways: A Review. Brain Plast. 2017;2(2):127–152. doi: 10.3233/BPL-160040. 29765853 PMC5928534

[7] van Praag H, Shubert T, Zhao C, Gage FH. Exercise enhances learning and hippocampal neurogenesis in mice. J Neurosci. 2005;25(38):8680–5; 16177036 10.1523/JNEUROSCI.1731-05.2005 PMC1360197

[8] Erickson KI, Voss MW, Prakash RS, Basak C, Szabo A, Chaddock L, Kim JS, Heo S, Alves H, White SM, Wojcicki TR, Mailey E, Vieira VJ, Martin SA, Pence BD, Woods JA, McAuley E, Kramer AF. Exercise training increase size of hippocampus and improves memory. Proc Natl Acad Sci U S A. 2011;108(7):3017–22 21282661 10.1073/pnas.1015950108 PMC3041121

[9] Piotrowicz Z, Chalimoniuk M, Płoszczyca K, Czuba M, Langfort J. Acute normobaric hypoxia does not affect the simultaneous exercise-induced increase in circulating BDNF and GDNF in young healthy men: A feasibility study PLoS One. 2019;14(10):e0224207. 31644554 10.1371/journal.pone.0224207 PMC6808427

[10] Meeusen R, De Meirleir K. Exercise and brain neurotransmission. Sports Med. 1995; 20(3):160–88. 8571000 10.2165/00007256-199520030-00004

[11] Noakes TD. Physiological models to understand exercise fatigue and the adaptations that predict or enhance athletic performance. Scand J Med Sci Sports. 2000;10(3):123–45. doi:10.1034/j.1600-0838.2000.010003123.x 10843507

[12] Noakes TD, Peltonen JE, Rusko HK. Evidence that a central governor regulates exercise performance during acute hypoxia and hyperoxia. J Exp Biol. 2001;204(Pt 18):3225–34. doi:10.1242/jeb.204.18.3225. 11581338

[13] Rothman DL, Behar KL, Hyder F, Shulman RG. In vivo NMR studies of the glutamate neurotransmitter flux and neuroenergetics: implications for brain function. Annu Rev Physiol. 2003;65:401–27. doi:10.1146/annurev.physiol.65.092101.142131. 12524459

[14] Kanamori K, Ross BD. Kinetics of glial glutamine efflux and the mechanism of neuronal uptake studied in vivo in mildly hyperammonemic rat brain. J Neurochem. 2006;99:1103–13. 17081141 10.1111/j.1471-4159.2006.04152.x

[15] Hertz L, Rothman DL. Glucose, Lactate, β-Hydroxybutyrate, Acetate, GABA, and Succinate as Substrates for Synthesis of Glutamate and GABA in the Glutamine-Glutamate/GABA Cycle. Adv Neurobiol. 2016;13:9–42. doi: 10.1007/978-3-319-45096-4_2. 27885625

[16] Yüksel C, Öngür D. Magnetic resonance spectroscopy studies of glutamate-related abnormalities in mood disorders. Biol Psychiatry. 2010;68:785–794. doi:10.1016/j.biopsych.2010.06.016. 20728076 PMC2955841

[17] Duarte JMN, Xin L. Magnetic resonance spectroscopy in schizophrenia: Evidence for glutamatergic dysfunction and impaired energy metabolism. Neurochem Res. 2019;44:102–116. doi:10.1007/s11064-018-2521-z. 29616444 PMC6345729

[18] Holschneider DP, Yang J, Guo Y, Maarek JM. Reorganization of functional brain maps after exercise training: Importance of cerebellar-thalamic-cortical pathway. Brain Res. 2007;1184:96–107. 17964551 10.1016/j.brainres.2007.09.081 PMC2692362

[19] Basso JC, Suzuki WA. The effects of acute exercise on mood, cognition, neurophysiology, and neurochemical pathways: A review1. Brain Plast. 2017;2:127–152. doi: 10.3233/BPL160040. 29765853 PMC5928534

[20] Maddock RJ, Casazza GA, Buonocore MH, Tanase C. Vigorous exercise increases brain lactate and Glx (glutamate+glutamine): a dynamic 1H-MRS study. Neuroimage. 2011;57:1324–1330. doi:10.1016/j.neuroimage. 21640838

[21] Maddock RJ, Casazza GA, Fernandez DH, Maddock MI. Acute Modulation of Cortical Glutamate and GABA Content by Physical Activity. J Neurosci. 2016;36:2449–2457. doi:10.1523/JNEUROSCI.3455-15.2016 26911692 PMC6705493

[22] Rasmussen P, Wyss MT, Lundby C. Cerebral glucose and lactate consumption during cerebral activation by physical activity in humans. FASEB J. 2011;25:2865–2873. doi:10.1096/fj.11-183822. 21602451

[23] Dennis A, Thomas AG, Rawlings NB, et al. An ultra-high field magnetic resonance spectroscopy study of post exercise lactate, glutamate and glutamine change in the human brain. Front Physiol. 2015;6:351. doi:10.3389/fphys.2015.00351. 26732236 PMC4681779

[24] Culham JC, Kanwisher NG. Neuroimaging of cognitive functions in human parietal cortex. Curr Opin Neurobiol. 2001;11(2):157–63. doi:10.1016/s0959-4388(00)00191-4. 11301234

[25] Sampaio-Baptista C, Scholz J, Jenkinson M, Thomas AG, Filippini N, Smit G, Douaud G, Johansen-Berg H. Gray matter volume is associated with rate of subsequent skill learning after a long term training intervention. Neuroimage. 2014;96:158–266. doi:10.1016/j.neuroimage.2014.03.056. 24680712 PMC4075341

[26] Vilcant V, Zeltser R. Treadmill Stress Testing. 2021 Jul 26. In: StatPearls[Internet]. Treasure Island (FL): StatPearls Publishing; 2021 Jan–. PMID:. 29763078

[27] Provencher SW. Estimation of metabolite concentrations from localized in vivo proton NMR spectra. Magn Reson Med. 1993;30(6):672–9. 8139448 10.1002/mrm.1910300604

[28] Pouwels PJ, Frahm J. Regional metabolite concentrations in human brain as determined by quantitative localized proton MRS. Magn Reson Med. 1998;39(1):53–60. doi: 10.1002/mrm.1910390110. 9438437

[29] Rivera-Brown AM, Frontera WR. Principles of exercise physiology: responses to acute exercise and long-term adaptations to training. PM R. 2012;4(11):797–804. 23174541 10.1016/j.pmrj.2012.10.007

[30] Hughes DC, Ellefsen S, Baar K. Adaptations to endurance and strength training. Cold Spring Harb Perspect Med. 2018;8(6):a029769. 28490537 10.1101/cshperspect.a029769 PMC5983157

[31] Cotman CW, Engesser-Cesar C. Exercise Enhances and Protects Brain Function. Exerc Sport Sci Rev. 2002;30:75–79. doi: 10.1097/00003677-200204000-00006 11991541

[32] Ludyga S., Gerber M., Brand S., Holsboer-Trachsler E., Puhse U. Acute Effects of Moderate Aerobic Exercise on Specific Aspects of Executive Function in Different Age and Fitness Groups: A Meta-Analysis. Psychophysiology. 2016;53:1611–1626. doi: 10.1111/psyp.12736 27556572

[33] Bengt Kayser. (Voluntary exercise starts and ends in the brain. Eur J Appl Physiol. 2003;90(3-4):411–9. doi: 10.1007/s00421-003-0902-7. 12883902

[34] Fonnum F, Hassel B. Glutamate synthesis, matabolism and uptake. (in:) CNS Neurotransmitters and Neuromodulators: Glutamate. Ed. TW Stone. CRC Press, Boca Raton 1995, Chapter 2, pp. 19–34.

[35] Erecińska M, Silver IA. Metabolism and role of glutamate in mammalian brain. Prog Neurobiol. 1990;35(4):245–96. doi: 10.1016/0301-0082(90)90013-7. 1980745

[36] Guillaud L, Dimitrov D, Takahashi T. Presynaptic morphology and vesicular composition determine vesicle dynamics in mouse central synapses. Elife. 2017;6:e24845. doi: 10.7554/eLife.24845. 28432787 PMC5423771

[37] Meldrum BS. Glutamate as a neurotransmitter in the brain: review of physiology and pathology. J Nutr. 2000 Apr;130(4S Suppl):1007S–15S. doi:10.1093/jn/130.4.1007S. 10736372

[38] Berl S, Clarke DD. Cerebral Glutamine/ Glutamate Interrelationships and Metabolic Compartmentation. In: Häussinger D, Sies H. (eds) Glutamine Metabolism in Mammalian Tissues. Springer. Berlin: Heidelberg; 1984. doi: 10.1007/978-3-642-69754-8_14

[39] Cooper AJ, Jeitner TM. Central Role of Glutamate Metabolism in the Maintenance of Nitrogen Homeostasis in Normal and Hyperammonemic Brain. Biomolecules. 2016;6(2):16. doi: 10.3390/biom6020016. 27023624 PMC4919911

[40] Guezennec CY, Abdelmalki A, Serrurier B, Merino D, Bigard X, Berthelot M, Pierard C, Peres M. Effects of prolonged exercise on brain ammonia and amino acids. Int J Sports Med. 1998;19(5):323–7. doi: 10.1055/s-2007-971925. 9721055

[41] van Hall G, Strømstad M, Rasmussen P, Jans O, Zaar M, Gam C, Quistorff B, Secher NH, Nielsen HB. Blood lactate is an important energy source for the human brain. J Cereb Blood Flow Metab. 2009;29(6):1121–9. doi: 10.1038/jcbfm.2009.35. 19337275

[42] Dager SR, Marro KI, Richards TL, Metzger GD. Localized magnetic resonance spectroscopy measurement of brain lactate during intravenous lactate infusion in healthy volunteers. Life Sci. 1992;51(12):973–85. doi: 10.1016/0024-3205(92)90404-d. 1325588

[43] Koush Y, de Graaf RA, Jiang L, Rothman DL, Hyder F. Functional MRS with J-edited lactate in human motor cortex at 4 T. Neuroimage. 2019;184:101–108. doi: 10.1016/j.neuroimage.2018.09.008. 30201463

[44] Schubert F, Gallinat J, Seifert F, Rinneberg H. Glutamate concentrations in human brain using single voxel proton magnetic resonance spectroscopy at 3 Tesla. Neuroimage. 2004;21(4):1762–71. doi: 10.1016/j.neuroimage.2003.11.014. 15050596

[45] Pradhan S, Bonekamp S, Gillen JS, Rowland LM, Wijtenburg SA, Edden RA, Barker PB. Comparison of single voxel brain MRS AT 3T and 7T using 32-channel head coils. Magn Reson Imaging. 2015;33(8):1013–8. doi: 10.1016/j.mri.2015.06.003. 26117693 PMC4549223

[46] Pfeuffer J, Tkác I, Provencher SW, Gruetter R. Toward an in vivo neurochemical profile: quantification of 18 metabolites in short-echo-time (1)H NMR spectra of the rat brain. J Magn Reson. 1999;141(1):104–20. doi: 10.1006/jmre.1999.1895. 10527748

[47] Rowland BC, Liao H, Adan F, Mariano L, Irvine J, Lin AP. Correcting for Frequency Drift in Clinical Brain MR Spectroscopy. J Neuroimaging. 2017;27(1):23–28. doi: 10.1111/jon.12388 27601075

