# Supplementary material for: Increased brain 1H-MRS glutamate and lactate signals following maximal aerobic capacity exercise in young healthy males: an exploratory study
Source: Biol Sport. 2022 Sep 15;40(3):665–73. doi: 10.5114/biolsport.2023.118335 (PMC10286605; doi:10.5114/biolsport.2023.118335)
Supplement: Increased brain 1H-MRS glutamate and lactate signals following maximal aerobic capacity exercise in young healthy males: an exploratory study [file JBS-40-118335-s1.pdf]

Supplemental material

Table S1. Spectral quality for each metabolite.

	NE Group		E Group		p Value
	M	SD	M	SD	
Snr	61.14	4.96	64.57	4.31	0.062
FWHM [Hz]	5.68	1.37	5.69	0.99	0.933
CRLB [%] Cr+PCr	1.93	0.27	1.93	0.26	> 0.99
CRLB [%] NAA	2.00	0.00	2.00	0.50	> 0.99
CRLB [%] NAA+NAAG	2.17	0.39	2.07	0.48	0.797
CRLB [%] GPC+PCh	2.75	0.45	2.64	0.50	0.574
CRLB [%] Ins	3.75	0.45	3.89	0.33	0.509
CRLB [%] GPC	6.08	5.79	8.71	8.99	0.946
CRLB [%] NAAG	17.8	6.80	16.67	3.75	0.634

Note: Values are mean and standard deviations. Data collected at rest (NE group) and exercise (E group). FWHM - Full width at half maximum herc [Hz] , CRLB - cramer-rao lower bound percentage [%] , Cr+PCr - creatine and phosphocreatine, NAA - N-acetylaspartate, NAA+NAAG - N-acetylaspartate and N-acetylaspartylglutamate, GPC+PCh - glycerophosphocholine and phosphocholine, Ins - inositol, GPC - glycerophosphocholine, NAAG - acetylaspartylglutamate.
